# Simultaneous Raman and infrared spectroscopy: a novel combination for studying bacterial infections at the single cell level†

**DOI:** 10.1039/d2sc02493d

**Published:** 2022-06-29

**Authors:** Cassio Lima, Shwan Ahmed, Yun Xu, Howbeer Muhamadali, Christopher Parry, Rachel J. McGalliard, Enitan D. Carrol, Royston Goodacre

**Affiliations:** Centre for Metabolomics Research, Department of Biochemistry and Systems Biology, Institute of Systems, Molecular and Integrative Biology, University of Liverpool Liverpool L69 7ZB UK roy.goodacre@liverpool.ac.uk; Department of Clinical Infection, Microbiology and Immunology, Institute of Infection, Veterinary and Ecological Sciences, University of Liverpool Liverpool L69 7BE UK; Department of Clinical Sciences, Liverpool School of Tropical Medicine Liverpool L3 5QA UK; Department of Environment and Quality Control, Kurdistan Institution for Strategic Studies and Scientific Research Kurdistan Region Iraq

## Abstract

Sepsis is a life-threatening clinical condition responsible for approximately 11 million deaths worldwide. Rapid and accurate identification of pathogenic bacteria and its antimicrobial susceptibility play a critical role in reducing the morbidity and mortality rates related to sepsis. Raman and infrared spectroscopies have great potential to be used as diagnostic tools for rapid and culture-free detection of bacterial infections. Despite numerous reports using both methods to analyse bacterial samples, there is to date no study collecting both Raman and infrared signatures from clinical samples simultaneously due to instrument incompatibilities. Here, we report for the first time the use of an emerging technology that provides infrared signatures *via* optical photothermal infrared (O-PTIR) spectroscopy and Raman spectra simultaneously. We use this approach to analyse 12 bacterial clinical isolates including six isolates of Gram-negative and six Gram-positive bacteria commonly associated with bloodstream infection in humans. To benchmark the single cell spectra obtained by O-PTIR spectroscopy, infrared signatures were also collected from bulk samples *via* both FTIR and O-PTIR spectroscopies. Our findings showed significant similarity and high reproducibility in the infrared signatures obtained by all three approaches, including similar discrimination patterns when subjected to clustering algorithms. Principal component analysis (PCA) showed that O-PTIR and Raman data acquired simultaneously from bulk bacterial isolates displayed different clustering patterns due to the ability of both methods to probe metabolites produced by bacteria. By contrast, signatures of microbial pigments were identified in Raman spectra, providing complementary and orthogonal information compared to infrared, which may be advantageous as it has been demonstrated that certain pigments play an important role in bacterial virulence. We found that infrared spectroscopy showed higher sensitivity than Raman for the analysis of individual cells. Despite the different patterns obtained by using Raman and infrared spectral data as input for clustering algorithms, our findings showed high data reproducibility in both approaches as the biological replicates from each bacterial strain clustered together. Overall, we show that Raman and infrared spectroscopy offer both advantages and disadvantages and, therefore, having both techniques combined in one single technology is a powerful tool with promising applications in clinical microbiology.

## Abstract 2

O-PTIR was used for simultaneous collection of infrared and Raman spectra from clinical pathogens associated with bloodstream infections.

## Graphical abstract

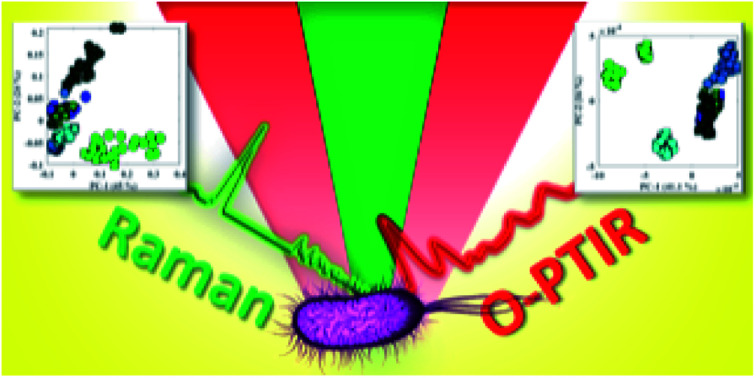

## Introduction

Sepsis is a life-threatening clinical condition defined as an “organ dysfunction caused by a dysregulated host response to infection” according to the Third International Consensus (Sepsis-3).^1^ Bacterial sepsis is a leading cause of death in both developed and developing nations, accounting for approximately 11 million deaths every year, which represents 19.7% of all deaths worldwide.^2,3^ Rapid and accurate identification of bacteria causing the infection, as well as its antimicrobial susceptibility, play a critical role in the timely diagnosis and treatment of bacterial infections including sepsis.^4,5^ However, current diagnostic methods for bacterial typing require time-consuming sample culturing protocols and thus, broad-spectrum antimicrobial are often prescribed as first-line medications as soon as possible in order to avoid detrimental outcomes caused by delayed treatment.^5,6^ Unnecessary antimicrobial treatment is the leading cause of the development of multidrug-resistant pathogens, which have dramatically increased worldwide over the years.^7^ The Centres for Disease Control and Prevention in the US estimates that 30% of patients with bacterial infections are treated with antimicrobials inappropriately.^5,7^ If actions are not taken, it is estimated that antimicrobial resistance will be responsible for around ten million deaths annually by 2050.^8^ New methods for rapid and culture-free detection of bacterial infections may help to reduce morbidity and mortality as well as preventing the emergence of resistant strains during treatment.

Vibrational spectroscopic techniques, represented mainly by Raman and infrared spectroscopies, have been widely used to study bacteria by interrogating their chemical and physicochemical properties in a non-invasive, non-destructive, and label-free manner.^4,9–11^ Both methods provide molecular information based on bond-specific chemical signatures and have been extensively applied as whole-organism fingerprinting tools to study the effects of antimicrobial agents in bacteria,^12–14^ classification and identification of microorganisms,^4,15,16^ and biofilms.^17–19^ Although these two methods are used for similar purposes, *i.e.*, to probe the internal motion of atoms from chemical species in molecules,^20^ Raman and infrared spectroscopies operate in completely different ways. Infrared spectroscopy is based on the absorption of infrared radiation by molecular vibrations from bonds that possess an electric dipole moment that can change by atomic displacement.^20,21^ By contrast, Raman spectroscopy is based on the inelastic scattering of light, in which the incident photons from a monochromatic source excite the molecules to virtual energy states and then, are scattered with either higher or lower energies compared to their original energy.^10,20,22^ This shift in energy corresponds to a Raman shift and like infrared is associated with the chemical structure of molecules in the sample. Due to these different principles, these two techniques are considered complementary as they offer advantages and disadvantages to study certain types of samples when compared to each other.^10^

Raman and infrared spectroscopies are often used together for the comprehensive study of bacteria. Nicolaou *et al.* used both techniques for the detection and enumeration of *Staphylococcus aureus* in milk,^23^ as well as to study the growth interaction between *S. aureus* and *Lactococcus lactis*.^23^ Tugarova and co-workers used both methods to study selenium nanoparticles synthesised by the bacterium *Azospirillum thiophilum*.^24^ Tang *et al.* used Raman and FTIR signatures to discriminate mycobacteria and Gram-negative bacteria.^25^ Adamou employed both Raman and FTIR to investigate the chalcopyrite–bacteria interactions and jarosite biosynthesis. Atykyan and colleagues applied the two techniques to study the structural differences of bacterial cellulose in *Gluconacetobacter sucrofermentans*.^26^ However, a key feature of all of these studies is that they were performed on two separate instruments as simultaneous measurement of Raman and infrared signatures is a challenge in spectroscopy due to the difficulty of sharing light sources and optics in a conventional Raman and FTIR spectrometers,^27^ and especially the case when one considers bacteria are typically just 1–2 μm in size. Recently, a novel far-field optical instrument able to provide Raman and infrared signatures simultaneously has been developed. In this scheme, the infrared signatures are collected *via* optical photothermal infrared (O-PTIR) spectroscopy, an emerging technique that has been developed to provide infrared chemical maps with submicron spatial resolution in a rapid manner, therefore overcoming the poor spatial resolution achieved by a conventional FTIR spectrometer (3 to 30 μm).^28,29^ In O-PTIR spectroscopy, the photothermal response of a sample illuminated by a tunable mid-IR laser beam is probed by a visible CW laser,^28^ which also acts as the excitation source for acquiring a Raman spectrum from the same sample location at the same spatial resolution. This scheme has been successfully applied to acquire Raman and infrared spectra simultaneously from mammalian cells,^29,30^ single cell bacterium,^13,29^ nanocomposite interfaces,^31^ and atmospheric particles.^32^ Although O-PTIR and Raman spectroscopy have been simultaneously applied to analyse bacteria,^13,29^ previous studies used non-commercial instruments and were focused on analysing laboratory strains, which have different features such as low virulence compared to strains isolated from humans, therefore hindering its translation to clinical setting to study human samples. In this study we report for the first time the use of concomitant Raman and infrared spectroscopies using a commercial technology to differentiate 12 bacterial strains isolated from humans in order to demonstrate the advantages of combining both methods in one single technology to study pathogenic bacteria commonly associated with bloodstream infections.

## Results and discussion

### Infrared spectroscopy

**Fig. 1:**
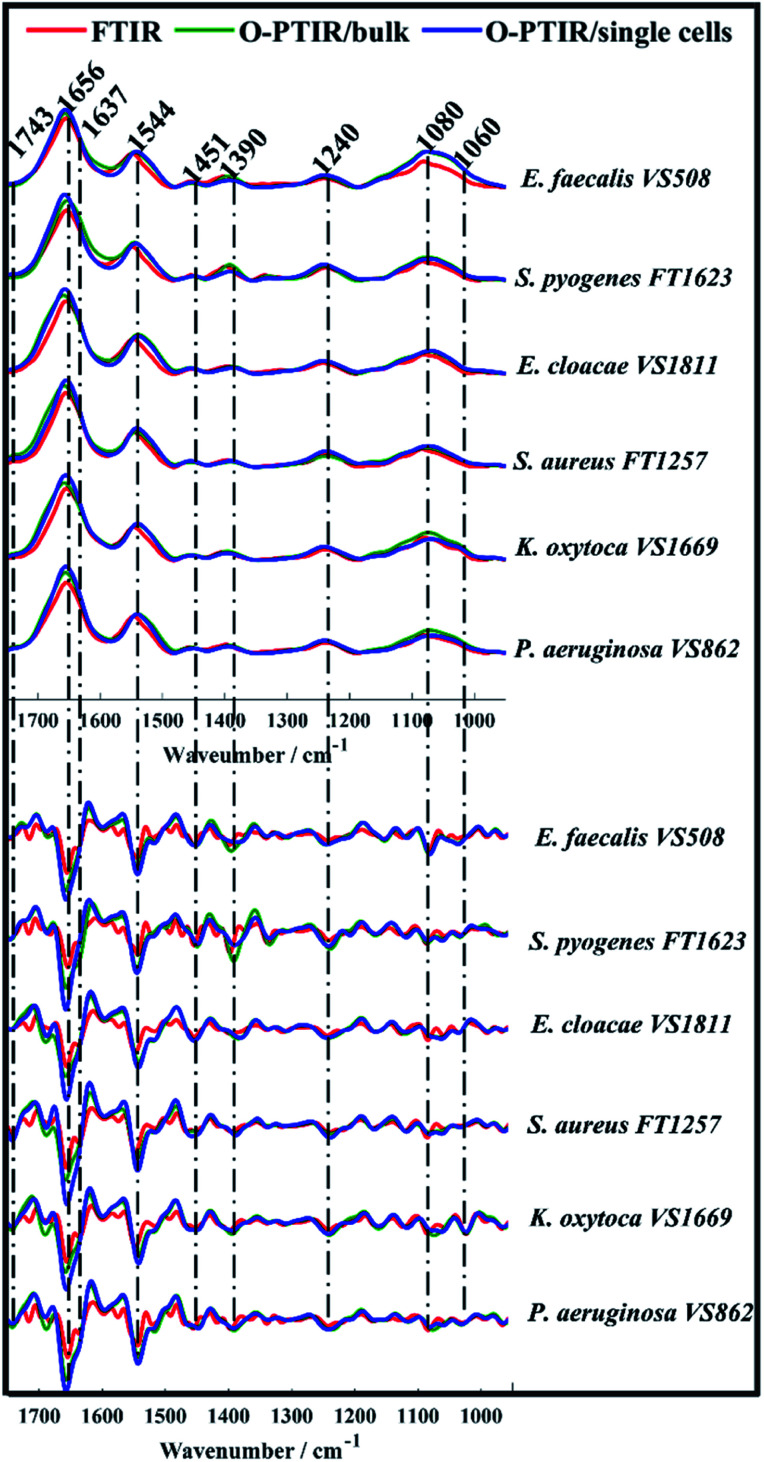
Average FTIR (red), O-PTIR/bulk (green), and O-PTIR/single cells (blue) spectra (top) and their second derivatives (bottom) collected from one strain of each bacterial species analysed in this study. The plots are offset for clarity. Key bands are highlighted (in cm^−1^).

O-PTIR spectra collected from bulk and single bacterial cells were compared to conventional FTIR spectra in order to evaluate the reproducibility of the infrared signatures provided by the three different approaches. Fig. 1 illustrates the average infrared spectra and their second derivatives acquired from one strain of each bacterial species analysed in this study. Spectral data from the remaining isolates are displayed in Fig. S1 (ESI†).

While single cell O-PTIR measurement is performed on a localised region of a single bacterium, FTIR and O-PTIR measurements acquired from bulk represent the average signal obtained from several bacteria within the beam area (millions in the case of FTIR where the interrogation beam is *ca*. 1 cm compared to 2–10 in O-PTIR where the probe is from a 532 nm laser). The spectra in Fig. 1 show significant similarity and high reproducibility in the infrared signatures obtained by all three approaches, which exhibited bands associated to vibrational modes related to the main macromolecules found in biological specimens such as proteins, lipids, nucleic acids, and carbohydrates. Spectral region over 1700–1400 cm^−1^ largely represents the amide region, which comprises amide I and II vibrations from backbone of proteins.^33^

The amide I vibration, peaking between 1690-1620 cm^−1^ arises mainly from the C=O stretching vibration from peptide bonds in proteins. This band is composed by the superposition of sub-bands associated to the secondary structure of proteins such as α-helices (1656 cm^−1^) and β-sheets (1637 cm^−1^).^28,33,34^ The signal intensity obtained from α-helices is usually superior than the signal collected from β-sheets in spectral data from bacteria, therefore, amide I band is centred around 1656 cm^−1^ due to the proximity of both peaks. In such cases, second derivatives are usually the preferred option to assess overlapped bands. According to Fig. 1, strong α-helices signatures (1656 cm^−1^) were observed in the second derivatives obtained by all three approaches. Interestingly, the signal acquired from β-sheets was more evident in the FTIR signatures than in the data obtained by O-PTIR, with the signal collected from bulk being slightly superior than that of individual cells. These differences may be related to the amount of material probed by each approach, since the polychromatic IR beam used in FTIR covers a large sample area with many individual cells in a community.

The Amide II band absorption is found at 1544 cm^−1^ and is attributed to the combination of N–H vibration with C–N stretching from peptide groups.^28,33,34^ The band peaking at 1743 cm^−1^ originates from the C=O ester groups from lipids including phospholipids, triglycerides and cholesterol.^34,35^

The peak at 1451 cm^−1^ is attributed to the bending vibration (scissoring) of acyl CH_2_ groups in lipids,^35^ whereas the band peaking at 1390 cm^−1^ arises from COO^−^ symmetric stretching of amino acid side chains and fatty acids.^34,35^ Bands peaking over the low wavenumber region in infrared spectra from bacteria are generally attributed to carbohydrates and nucleic acids. Bands associated to PO_2_^^−^^ asymmetric and symmetric stretching vibrations from phosphodiester bonds in nucleic acids are displayed in 1240 cm^−1^ and 1080 cm^−1^, respectively.^34,35^ The band peaking at 1060 cm^−1^ is associated to C–O–C symmetric stretching of polysaccharides.^19,35^

Spectral data collected from all bacterial isolates (Fig. 1 and S1†) displayed similar biochemical features with subtle changes in signal intensity, while appearance or disappearance of peaks were not observed. In order to examine the ability of FTIR and O-PTIR to discriminate the 12 bacterial isolates, the second derivatives calculated from spectra were subjected to principal component analysis (PCA).

**Fig. 2:**
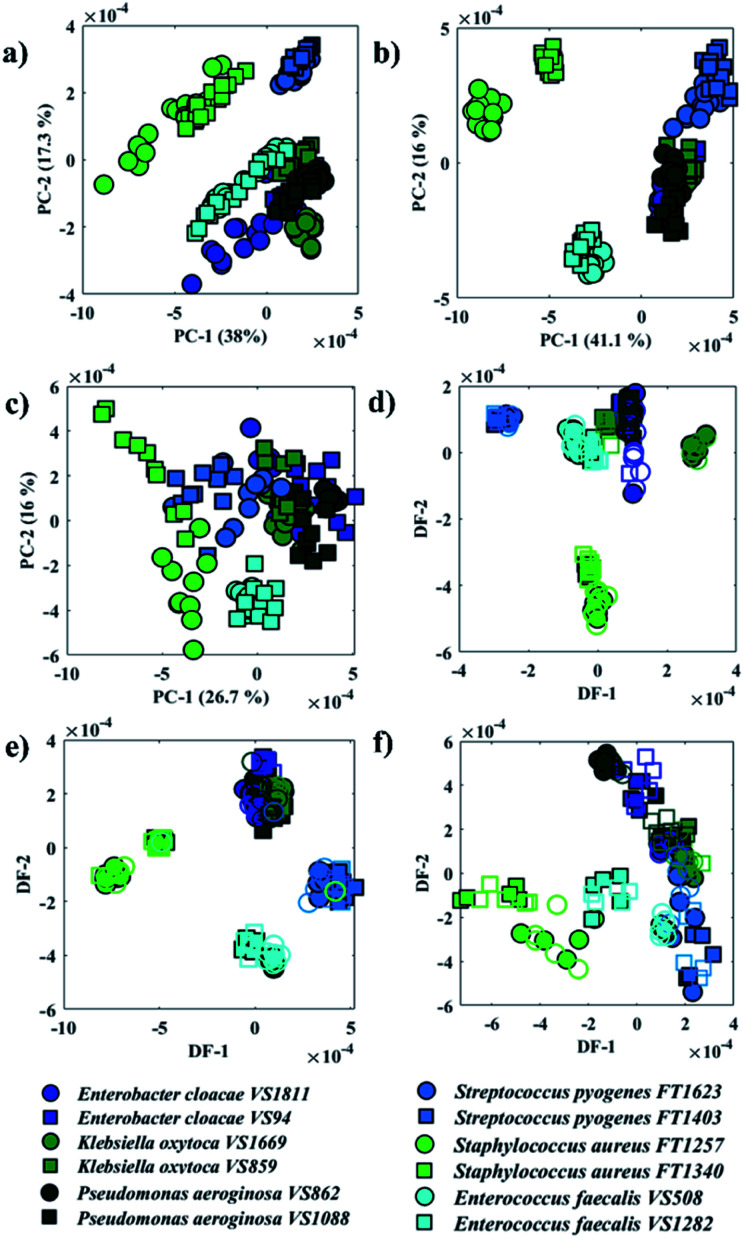
PCA (a–c) and PC-DFA (d–f) scores plot of FTIR (a and d), O-PTIR/bulk (b and e), and O-PTIR/single cells (c and f) data. The same species are plotted in the same colours and the different symbols represent one of the two sub-species. Filled symbols represent the training set, while empty symbols are the testing set.

Fig. 2a–c illustrates PCA scores plot of the FTIR, O-PTIR (bulk), and O-PTIR (single cells) data, respectively. In Fig. 2a, scores from *S. aureus*, *E. faecalis* and *E. cloacae* VS1811 isolates were grouped on the negative side of the PC-1 axis, while all other isolates were clustered on the positive side. In PC-2 axis, *S. aureus* and *S. pyogenes* isolates grouped on the positive side and the remaining isolates clustered on the negative axis of PC-2. PCA scores obtained from O-PTIR (bulk) (Fig. 2b) displayed similar discrimination pattern compared to FTIR data (Fig. 2a), with scores from Gram-negative bacteria (*E. cloacae*, *K. oxytoca* and *P. aeruginosa*) isolates forming a tight cluster. In Fig. 2c, PCA scores plot of O-PTIR (single cells) illustrated the same cluster formed by Gram-negative bacteria (*E. cloacae*, *K. oxytoca*, and *P. aeruginosa*) on the positive side of PC-1 axis, whereas the other isolates were grouped along negative PC-1 values.

Although all three approaches have illustrated similar clustering patterns, the data reproducibility, *i.e.* the distance between scores from the same strain, considerably varies in the three methods. O-PTIR (single cells) showed the lowest reproducibility compared to FTIR and O-PTIR (bulk) data. This is expected due to the ability of the method to probe inter-individual variations in bacterial metabolism and highlights the potential applications of O-PTIR to generate population level information from single cells. As such this is an exciting emerging technology to study the interaction between microorganisms as well as the role of each individual in microbial communities. Interestingly, PCA scores from O-PTIR (bulk) showed higher reproducibility compared to FTIR, as the scores of each strain were grouped close to each other, while FTIR scores were grouped in a more dispersed pattern. In some cases, it is even possible to observe a trend on the FTIR scores pattern such as in *S. aureus* and *E. faecalis*, which suggests varying quantities of a biochemical component in the spectra. These findings were unexpected as FTIR provides the average signal from a larger area compared to the spot probed in O-PTIR (bulk). Thus, in order to improve the reproducibility and, therefore, the ability of each approach to identify each bacterial strain correctly, the principal components (PCs) were used as input data for DFA (PC-DFA) in order to minimise the variance within classes, while maximising the variance between classes.^16^ PC-DFA is a supervised method, *i.e.* the algorithm takes into consideration an *a priori* knowledge about the isolates. Thus, 50% of the spectra from each strain (class) were randomly selected and used to generate the model (training set), while the remaining data (testing set) were projected into the PC-DFA space to validate the model as well as to test the occurrence of data overfitting.

**Fig. 3:**
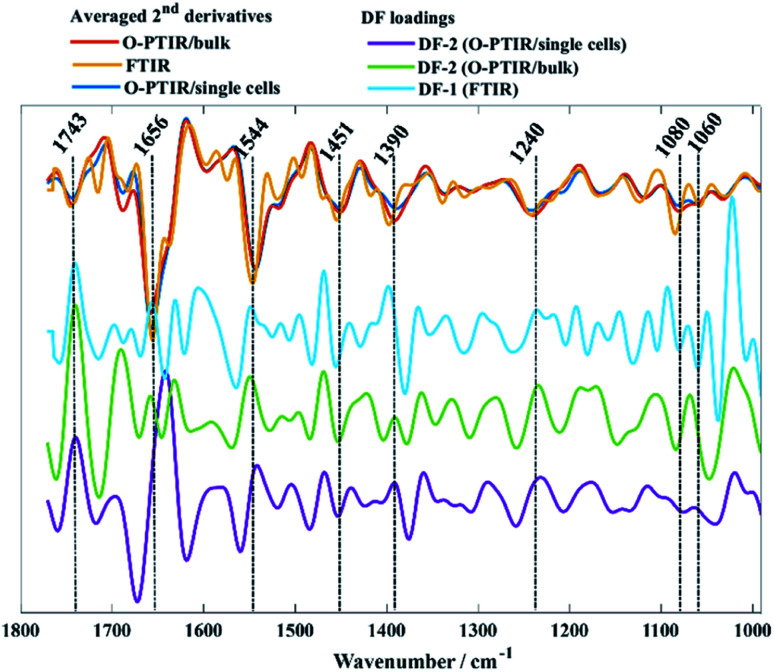
Averaged second derivatives from FTIR (yellow), O-PTIR/bulk (red), and O-PTIR/single cells (blue) from all bacterial isolates. DF-1 loadings from FTIR (cyan), DF-2 from O-PTIR/bulk (green) and DF-2 from O-PTIR/single cells (purple) associated to the PC-DFA scores plots displayed in Fig. 2d–f. The plots are offset for clarity.

Fig. 2d–f shows PC-DFA scores plot of the FTIR, O-PTIR (bulk), and O-PTIR (single cells) data, respectively. The reproducibility of the data was successfully enhanced in the DFA scores obtained in all three approaches, including the disappearance of trend patterns observed in the PCA scores from *S. aureus* and *E. faecalis* in FTIR data (Fig. 2a). Despite the improvement on the data reproducibility of O-PTIR (single cells) compared to its PCA scores plot (Fig. 2c), there is still considerable variability between the DFA scores from the same class, reinforcing the ability of O-PTIR to probe inter-individual variations in bacterial metabolism. Overall, DFA scores from training (filled symbols) and testing set (empty symbols) were clustered together, with only a few misclassifications, indicating that the clustering pattern obtained is due to bacterial phenotypes, and that such pattern is reproducible with no data overfitting. The cluster formed by Gram-negative bacterial isolates (*E. cloacae*, *K. oxytoca*, and *P. aeruginosa*) previously observed in the PCA scores (Fig. 2a–c) was grouped on the positive side of DF-1 from FTIR and on the positive side of DF-2 axis in the DFA scores obtained from O-PTIR data (both bulk and single cells). Interestingly, the Gram-positive isolates (*E. faecalis*, *S. aureus*, and *S. pyogenes*) grouped along the opposite side of the respective DFs but not in a tight cluster as depicted by *E. cloacae*, *K. oxytoca*, and *P. aeruginosa* isolates in Fig. 2d–e. Despite the disperse clustering pattern between these different bacterial species, isolates of the same species tended to group close to each other. Fig. 3 shows the DFA loadings from FTIR (DF-1) and O-PTIR (DF-2) measurements, which revealed significant similarities in the main vibrational modes that contribute to the separation of the clusters containing Gram-negative and Gram-positive bacterial isolates in each of the three different approaches.

### Raman spectroscopy

Raman spectra from single bacterial cells and bulk populations were collected simultaneously with infrared data from all bacterial isolates. However, the reproducibility of Raman signatures obtained from single cells of most bacterial isolates was quite poor (data not shown), so that we could not even reproduce most of the Raman peaks illustrated in Raman spectra acquired from bulk populations. The two strains of *S. pyogenes* (FT1623 and FT1403) and *E. faecalis* (VS1282 and VS508) were the only bacterial isolates that generated reproducible and reliable Raman spectra at single cell level (Fig. S2†). Acquiring Raman spectrum from a single cell bacterium is often challenging by spontaneous Raman spectroscopy due to its lower sensitivity compared to infrared spectroscopy as the mass of a single bacterium would be about one picogram.^36^ Besides that, illuminating a single cell bacterium with relatively high laser energies for a certain period of time increases the local temperature resulting in photochemical effects, which may alter the Raman signatures due to thermal damage or even induce total cell degradation due to carbonisation.

In such situations, other Raman-based approaches with higher sensitivity such as SERS is preferred for single cell analysis as it can provide enhancements of 10^3^–10^6^-fold compared to spontaneous Raman scattering.^15,22^ Thus, the present study focused on analysing Raman signatures acquired from bulk samples and not individual cells.

**Fig. 4:**
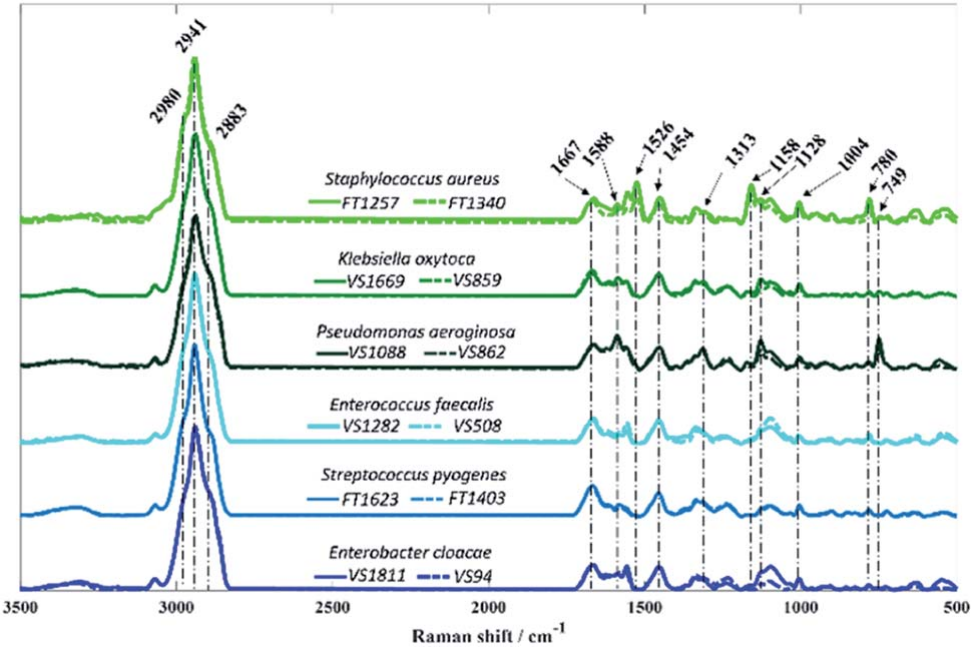
Average Raman spectra collected from bacterial isolates analysed in this study. Each colour represents a species, while the different sub-species are displayed in solid and dashed lines. The plots are offset for clarity.

In contrast to O-PTIR spectroscopy, where the spectral range is limited to the fingerprint region (1800–950 cm^−1^) due to the QCL wavelengths, Raman spectra were acquired over 3500–500 cm^−1^ from all bacterial isolates analysed in this study (Fig. 4).

As in infrared spectroscopy, the bands observed in Raman spectra are also associated with vibrations of the main molecular constituents in bacteria. While the infrared spectra from O-PTIR contained broad features that were largely highly similar biochemical signatures with subtle changes in band intensities, the Raman features obtained from some isolates exhibited highly distinct peaks compared to other isolates, as well as considerable differences in signal intensities.

Vibrational modes peaking in the high wavenumber region are attributed to CH– stretching vibrations, which exhibited similar bands in spectra acquired from all isolates. The band peaking at 2883 cm^−1^ is attributed to CH– symmetric stretching vibrations of CH_2_ in lipids, 2941 cm^−1^ is associated to CH– stretching in CH_3_ groups from lipids and proteins, whereas the peak seen in 2980 cm^−1^ is due to nucleic acid vibrations.^37^ Band peaking at 1667 cm^−1^ arises mainly from the C=O stretching vibration from backbone structure in proteins (amide I).^9^*P. aeruginosa* isolates are the only two strains displaying the band at 749 cm^−1^, which can be attributed to the pyrrole breathing mode in cytochromes.^38,39^ In addition to this, strong Raman peaks typical from cytochromes appear at 1128, 1313, and 1588 cm^−1^ in *P. aeruginosa* isolates.^38,39^ Raman spectra collected from *S. aureus* isolates presents two distinct bands peaking at 1158 and 1526 cm^−1^ resembling carotenoid staphyloxanthin bands, which are assigned to C–C stretching and C=C stretching vibrations, respectively.^40,41^ Staphyloxanthin is the pigment responsible for the golden colour in *S. aureus* and plays an important factor for the integrity of cell membrane as well as in the virulence of *S. aureus*.^40,42,43^ Although the band at 780 cm^−1^ displays a strong signal in *S. aureus* isolates, this peak is due to pyrimidine ring breathing and not to staphyloxanthin, as it has also been documented in unpigmented *S. aureus*.^40^ The band peaking at 1454 cm^−1^ originates from CH_2_/CH_3_ bending vibrations from lipids and proteins,^44^ while the peak at 1004 cm^−1^ is attributed to the phenylalanine ring breathing.^44^

**Fig. 5:**
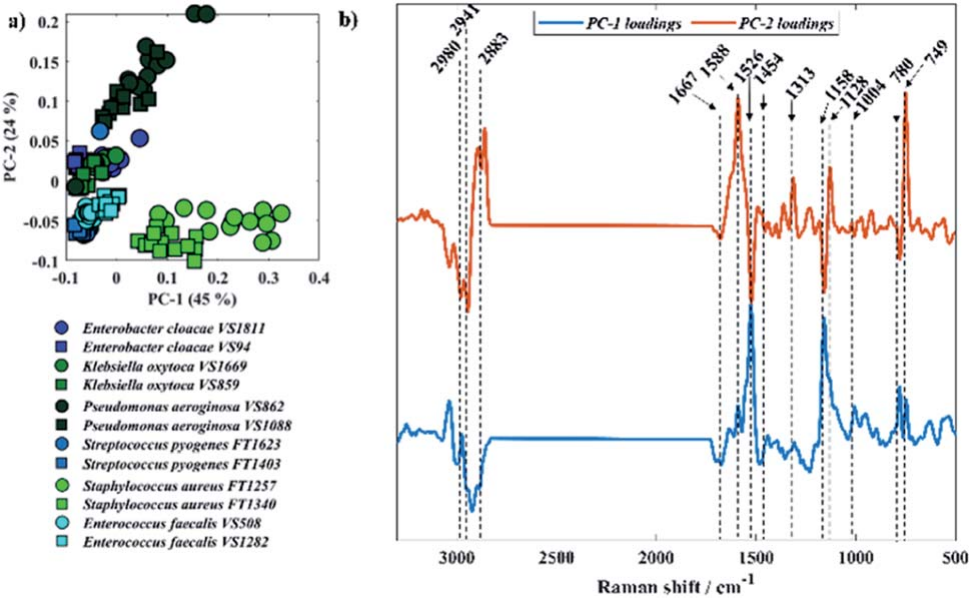
(a) PCA scores plot of Raman data, same species are plotted in similar colour and different symbols represent each one of the two sub-species. (b) PCA loadings associated to PC-1 and PC-2. The plots are offset for clarity.

Fig. 5 displays PCA results obtained for Raman spectra of all twelve bacterial isolates. In Fig. 5a, scores from *S. aureus*, *P. aeruginosa* VS862, and a few scores from *P. aeruginosa* VS1088 isolates were grouped on the positive axis of PC-1, while most of the other scores from *P. aeruginosa* VS1088 and the remaining isolates were clustered on the negative axis. Interestingly, the discrimination pattern achieved by PC-1 is mainly due to the pyrimidine ring breathing (780 cm^−1^) from *S. aureus* and bacterial pigments, as positive loadings associated to cytochromes from *P. aeruginosa* (1588, 1128, and 749 cm^−1^) and staphyloxanthin (1158 and 1526 cm^−1^) from *S. aureus* strains were documented. Scores from the two *P. aeruginosa* isolates grouped on different sides of PC-1, which suggests varying cytochrome content between the two strains, with higher content in the VS862 strain. Although both *S. aureus* isolates have been clustered on the same PC-1 axis, scores from both strains were also grouped in a gradient pattern, indicating increasing staphyloxanthin content along PC-1 axis, with higher content in the FT1257 strain. Interestingly, the discrimination pattern observed in PC-2 axis was similar to the grouping pattern illustrated in infrared spectroscopy, with Gram-negative strains (*E. cloacae*, *K. oxytoca*, and *P. aeruginosa*) grouping on the positive axis of PC-2, while Gram-positive bacteria (*S. aureus*, *S. pyogenes*, *E. faecalis*) clustered on the negative axis.

Clustering patterns obtained in infrared and Raman spectroscopies were different when compared to each other due to the different molecular species that were highlighted by the two different techniques. To explore this further, Procrustes analysis was performed in order to compare the similarity in the patterns generated by Raman spectroscopy and the three infrared spectroscopy approaches in a quantitative way. The similarity is measured based on the Procrustes error, which varies from 0 to 1; where 0 indicates identical patterns and 1 indicates completely different clustering patterns (Table 1). O-PTIR data from bulk and individual cells displayed the highest similarity level with a Procrustes error of 0.0163, which is expected due to the similar clustering observed in the PC-DFA scores (Fig. 2e–f). On the other hand, pairwise comparisons of Raman data with any of the three infrared-based approaches displayed the lowest similarity levels, with Procrustes errors varying between 0.3290-0.3520. These variations are mainly associated with the differences in the principles of these techniques and their ability to detect different groups of metabolites produced by these bacteria. Microorganisms, including bacteria, act like microbial cell factories due to the production of metabolites such as antibacterials,^45^ antifungals,^46^ vitamins,^47^ enzymes,^48^ and pigments.^41^ Although these metabolites may be infrared active, their infrared signatures have poor signal due to the low concentration compared to the other bacterial molecular constituents such as proteins, nucleic acids, carbohydrates, and lipids. On the other hand, some of these metabolites display molecular absorption bands compatible or near the wavelength used for excitation in Raman spectroscopy, resulting in the amplification of their Raman vibrations *via* resonance. As a result, the enhancement of the signatures from these ‘chromophore’ compounds will ultimately change the Raman spectra as well as the discrimination pattern achieved by each technique. Besides that, only the fingerprint region of infrared spectra (1800–950 cm^−1^) was used as input data to the multivariate statistical methods, whereas the input data used in Raman analysis included vibrations peaking over the high wavenumber region and thus, providing extra information. This limitation is due to the QCLs used in the spectrometer employed in our study, which can be overcome by the addition of a tunable OPO-pulsed infrared laser to cover the higher wavenumber region.^30^ Nevertheless these observed Procrustes errors are still statistically highly significant compared to the NULL distribution when subjected to Procrustean test (*p* < 0.0001).^49^ This suggests that the patterns of three IR based approaches are mostly similar to that of Raman data.

**Table 1:** Similarity between different datasets using Procrustes distance

PC-DFA scores	FTIR	Raman	O-PTIR/bulk	O-PTIR/single-cells
FTIR	—			
Raman	0.3520	—		
O-PTIR/bulk	0.1965	0.3353	—	
O-PTIR/single-cells	0.2184	0.3290	0.0163	—

## Experimental

### Growth conditions and sample preparation

Bacterial strains tested in this study consist of 12 bacteria isolated from clinical samples and identified at Alder Hey Children's NHS Foundation Trust according to standard diagnostic microbiology laboratory protocols in 2012 and 2017 (ethical approval granted by REC 19/SC/0306), and included six isolates of Gram-negative and six Gram-positive bacteria (Table 2). All bacterial isolates had been stored in glycerol at −80 °C since the time of isolation. The stored isolates were sub-cultured on brain heart infusion agar (BHI) at 37 °C for 24 h. After the incubation period, the bacteria were prepared by harvesting the biomass from the surface of each plate using sterile inoculating loops and resuspended in 1 mL of deionised water. Samples were washed in water 3 times in order to remove any residues from the media. Bacterial concentration was adjusted to an optical density (OD_600_) of 15.

**Table 2:** List of bacterial isolates including species and sub-species examined in this study

Bacterial strain	Original source
*Enterobacter cloacae* VS1811	Human faeces
*Enterobacter cloacae* VS94	Human faeces
*Klebsiella oxytoca* VS1669	Human blood
*Klebsiella oxytoca* VS859	Human faeces
*Pseudomonas aeruginosa* VS1088	Human blood
*Pseudomonas aeruginosa* VS862	Human blood
*Streptococcus pyogenes* FT1623	Human blood
*Streptococcus pyogenes* FT1403	Human blood
*Staphylococcus aureus* FT1257	Human blood
*Staphylococcus aureus* FT1340	Human blood
*Enterococcus faecalis* VS1282	Human blood
*Enterococcus faecalis* VS508	Human faeces

### FTIR spectroscopy

FTIR data were acquired from bulk populations following similar sample preparation and data collection protocols previously used by our group.^16^ 20 μL aliquots from each sample with an optical density of 15 (OD_600_) was transferred to silicon substrates (Bruker Ltd, Coventry, UK) and dried in an oven (50 °C) prior data collection. FTIR data were acquired in the mid-IR range (4000–600 cm^−1^), with 64 spectral co-adds and 4 cm^−1^ resolution, using an accessory designed for high-throughput measurements (HTS-XT) coupled to a Bruker Invenio FTIR spectrometer. A total number of 16 FTIR spectra were collected from each bacterial strain including 4 spectra acquired from 4 analytical replicates.

### O-PTIR and Raman spectroscopy

O-PTIR data were obtained from bulk populations and single cell bacterium. 2 μL aliquots from bulk samples (OD_600_ = 15) were spotted onto calcium fluoride (CaF_2_) substrates. For individual cell analysis, bacterial samples with OD_600_ of 15 was diluted in deionised water (1 : 1000) in order to obtain single cells, then 2 μL aliquots were spotted onto to CaF_2_ substrates. Samples were then dried in the oven. A mIRage infrared microscope (Photothermal Spectroscopy Corp., Santa Barbara, USA) was used to acquire single-point O-PTIR measurements in reflection mode (Cassegrain 40× objective (0.78 NA)) over the fingerprint mid-IR range (950–1800 cm^−1^; 2 cm^−1^ spectral resolution and 10 scans per spectrum) with the pump beam consisting of a tunable QCL device, whereas a continuous wave (CW) 532 nm laser was as probe beam. Raman scattering was detected simultaneously with infrared data from the same location on the sample after the photons passed through the Cassegrain objective into a Horiba iHR320 spectrometer equipped with a 600 groove per mm grating. Spectra were recorded over the range of 3500–500 cm^−1^, integration time of 10 s, 10 accumulations per spectrum, and spectral resolution of 4 cm^−1^. In total 15 infrared and Raman spectra were collected simultaneously from bulk populations of cells for each bacterial strain. For single cell analysis, 30 infrared and Raman spectra (3 spectra per cell, 10 cells in total) were collected from single individual bacterial cells.

### Data analysis

All statistical analysis was carried out in MATLAB version 2019a (The Mathworks Inc., Natwick, USA). Spectral data collected in all approaches were subjected to principal component analysis (PCA) as well as to principal component-discriminant function analysis (PC-DFA) as clustering algorithms. Before PCA and PC-DFA spectra were submitted to baseline correction using an asymmetric least squares algorithm, smoothed *via* a Savitzky–Golay filter using a polynomial of second order in a 7-point window, and vector normalised. For comparison of clustering patterns from the different spectroscopies Procrustes transformations were performed using a number of 3 DFs for each data set. All code used is available *via* Github (https://github.com/Biospec/).

## Conclusions

Here, we report for the first time the use of simultaneous Raman and infrared spectroscopies to study 12 clinically important bacteria isolated from humans who were suffering from sepsis. Infrared signatures were collected from bulk samples *via* FTIR and O-PTIR spectroscopies, while infrared data from individual bacterial cells was obtained by O-PTIR spectroscopy only. Although single cell measurements are performed on localised areas of a single bacterium, compared to bulk FTIR and O-PTIR measurements from several bacteria within the beam, our findings showed significant similarity and high reproducibility in the infrared signatures obtained by all three approaches including similar discrimination patterns when subjected to cluster analysis. These results highlight the potential applications of O-PTIR spectroscopy as an emerging technology to study microorganisms at single cell level, which has not been previously achieved by FTIR spectroscopy due to the poor spectral resolution provided by a conventional FTIR spectrometer. We also report, for the first time, the use of O-PTIR technology to acquire infrared and Raman spectral data simultaneously from bulk bacterial isolates samples, which displayed different clustering patterns when subjected to PCA due to the ability of Raman spectroscopy to probe pigments produced by bacteria such as staphyloxanthin and cytochromes. The superior ability of Raman spectroscopy to identify pigments may be advantageous regarding its use to study pathogenic bacteria as it has been demonstrated that several pigments promote microbial virulence. Despite the different patterns obtained by using Raman and infrared spectral data as input for clustering algorithms, our findings showed high data reproducibility in both approaches as the biological replicates from each bacterial strain clustered together. Thus, Raman and infrared spectroscopy offer both advantages and disadvantages, therefore, having both techniques combined in one single technology is a promising tool for clinical microbiology.

## Author contributions

Conceptualisation: RG, RM, EC; experiments: SA and CL; data analysis: CL and YX; supervision: RG, HM, RM, CP and EC; writing: CL; review & editing: all authors.

## Conflicts of interest

There are no conflicts to declare.

## Data availability

The data that support the findings of this study are available from the first author (CL) on request.

## Supplementary Material

SC-013-D2SC02493D-s001

## References

[cit1] Singer M., Deutschman C. S., Seymour C. W., Shankar-Hari M., Annane D., Bauer M., Bellomo R., Bernard G. R., Chiche J. D., Coopersmith C. M., Hotchkiss R. S., Levy M. M., Marshall J. C., Martin G. S., Opal S. M., Rubenfeld G. D., van der Poll T., Vincent J. L., Angus D. C. (2016). JAMA.

[cit2] Jarczak D., Kluge S., Nierhaus A. (2021). Front Med.

[cit3] Rudd K. E., Johnson S. C., Agesa K. M., Shackelford K. A., Tsoi D., Kievlan D. R., Colombara D. V., Ikuta K. S., Kissoon N., Finfer S., Fleischmann-Struzek C., Machado F. R., Reinhart K. K., Rowan K., Seymour C. W., Watson R. S., West T. E., Marinho F., Hay S. I., Lozano R., Lopez A. D., Angus D. C., Murray C. J. L., Naghavi M. (2020). Lancet.

[cit4] AlMasoud N., Muhamadali H., Chisanga M., AlRabiah H., Lima C. A., Goodacre R. (2021). Analyst.

[cit5] Ho C. S., Jean N., Hogan C. A., Blackmon L., Jeffrey S. S., Holodniy M., Banaei N., Saleh A. A. E., Ermon S., Dionne J. (2019). Nat. Commun..

[cit6] Martinez M. L., Plata-Menchaca E. P., Ruiz-Rodriguez J. C., Ferrer R. (2020). J. Thorac. Dis..

[cit7] Fleming-Dutra K. E., Hersh A. L., Shapiro D. J., Bartoces M., Enns E. A., File, Jr. T. M., Finkelstein J. A., Gerber J. S., Hyun D. Y., Linder J. A., Lynfield R., Margolis D. J., May L. S., Merenstein D., Metlay J. P., Newland J. G., Piccirillo J. F., Roberts R. M., Sanchez G. V., Suda K. J., Thomas A., Woo T. M., Zetts R. M., Hicks L. A. (2016). JAMA.

[cit8] ÓNeill J. , Antimicrobial resistance: tackling a crisis for the health and wealth of nations, https://bit.ly/36u4nKB, (accessed October, 25, 2021)

[cit9] Lu X., Al-Qadiri H. M., Lin M., Rasco B. A. (2011). Food Bioprocess Technol..

[cit10] Paraskevaidi M., Matthew B. J., Holly B. J., Hugh B. J., Thulya C. P. V., Loren C., StJohn C., Peter G., Callum G., Sergei K. G., Kamila K., Maria K., Kássio L. M. G., Pierre M.-H. L., Evangelos P., Savithri P., John A. A., Alexandra S., Marfran S., Josep S.-S., Gunjan T., Michael W., Bayden W. (2021). Appl. Spectrosc. Rev..

[cit11] Harrison J. P., Berry D. (2017). Front. Microbiol..

[cit12] Sharaha U., Rodriguez-Diaz E., Riesenberg K., Bigio I. J., Huleihel M., Salman A. (2017). Anal. Chem..

[cit13] Xu J., Li X., Guo Z., Huang W. E., Cheng J. X. (2020). Anal. Chem..

[cit14] Zhang M., Hong W., Abutaleb N. S., Li J., Dong P. T., Zong C., Wang P., Seleem M. N., Cheng J. X. (2020). Adv. Sci..

[cit15] Jarvis R. M., Goodacre R. (2004). Anal. Chem..

[cit16] Muhamadali H., Weaver D., Subaihi A., AlMasoud N., Trivedi D. K., Ellis D. I., Linton D., Goodacre R. (2016). Analyst.

[cit17] Wickramasinghe N. N., Hlaing M. M., Ravensdale J. T., Coorey R., Chandry P. S., Dykes G. A. (2020). Sci. Rep..

[cit18] Holman N., Miles R., Hao Z., Wozei E., Anderson L. M., Yang H. (2009). Anal. Chem..

[cit19] Quiles F., Humbert F., Delille A. (2010). Spectrochim. Acta, Part A.

[cit20] Diem M., Mazur A., Lenau K., Schubert J., Bird B., Miljkovic M., Krafft C., Popp J. (2013). J. Biophotonics.

[cit21] Baker M. J., Trevisan J., Bassan P., Bhargava R., Butler H. J., Dorling K. M., Fielden P. R., Fogarty S. W., Fullwood N. J., Heys K. A., Hughes C., Lasch P., Martin-Hirsch P. L., Obinaju B., Sockalingum G. D., Sule-Suso J., Strong R. J., Walsh M. J., Wood B. R., Gardner P., Martin F. L. (2014). Nat. Protoc..

[cit22] Lima C., Muhamadali H., Goodacre R. (2021). Annu. Rev. Anal. Chem..

[cit23] Nicolaou N., Xu Y., Goodacre R. (2011). Anal. Chem..

[cit24] Tugarova A. V., Mamchenkova P. V., Dyatlova Y. A., Kamnev A. A. (2018). Spectrochim. Acta, Part A.

[cit25] Tang M., McEwen G. D., Wu Y., Miller C. D., Zhou A. (2013). Anal. Bioanal. Chem..

[cit26] Atykyan N., Revin V., Shutova V. (2020). AMB Express.

[cit27] Hashimoto K., Badarla V. R., Kawai A., Ideguchi T. (2019). Nat. Commun..

[cit28] Lima C., Muhamadali H., Xu Y., Kansiz M., Goodacre R. (2021). Anal. Chem..

[cit29] Li X., Zhang D., Bai Y., Wang W., Liang J., Cheng J. X. (2019). Anal. Chem..

[cit30] Spadea A., Denbigh J., Lawrence M. J., Kansiz M., Gardner P. (2021). Anal. Chem..

[cit31] Wang A. J., Dillon E. P., Maharjan S., Liao K. S., McElhenny B. P., Tong T., Chen S., Bao J., Curran S. A. (2021). Adv. Mater. Interfaces.

[cit32] Olson N. E., Xiao Y., Lei Z., Ault A. P. (2020). Anal. Chem..

[cit33] Barth A. (2007). Biochim. Biophys. Acta.

[cit34] Naumann D. (2001). Appl. Spectrosc. Rev..

[cit35] Kardas M., Gozen A. G., Severcan F. (2014). Aquat. Toxicol..

[cit36] Loferer-Krössbacher M., Klima J., Psenner R. (1998). Appl. Environ. Microbiol..

[cit37] Lima C., Theron C. W., Muhamadali H., Kell D. B., Goodacre R. (2021). Clinical Spectroscopy.

[cit38] Hamada K., Fujita K., Smith N. I., Kobayashi M., Inouye Y., Kawata S. (2008). J. Biomed. Opt..

[cit39] Okada M., Smith N. I., Palonpon A. F., Endo H., Kawata S., Sodeoka M., Fujita K. (2012). Proc. Natl. Acad. Sci. U. S. A..

[cit40] Ayala O. D., Wakeman C. A., Pence I. J., Gaddy J. A., Slaughter J. C., Skaar E. P., Mahadevan-Jansen A. (2018). ACS Infect. Dis..

[cit41] Jehlicka J., Edwards H. G., Oren A. (2014). Appl. Environ. Microbiol..

[cit42] Ayala O. D., Wakeman C. A., Pence I. J., O'Brien C. M., Werkhaven J. A., Skaar E. P., Mahadevan-Jansen A. (2017). Anal. Methods.

[cit43] Liu G. Y., Nizet V. (2009). Trends Microbiol..

[cit44] Chisanga M., Muhamadali H., McDougall D., Xu Y., Lockyer N., Goodacre R. (2021). Analyst.

[cit45] Kong D., Wang X., Nie J., Niu G. (2019). Front. Microbiol..

[cit46] De Muynck C., Leroy A. I., De Maeseneire S., Arnaut F., Soetaert W., Vandamme E. J. (2004). Microbiol. Res..

[cit47] Soto-Martin E. C., Warnke I., Farquharson F. M., Christodoulou M., Horgan G., Derrien M., Faurie J. M., Flint H. J., Duncan S. H., Louis P. (2020). mBio.

[cit48] Nigam P. S. (2013). Biomolecules.

[cit49] Jackson D. A. (1995). Écoscience.

