# Supplementary material for: Simultaneous Raman and infrared spectroscopy: a novel combination for studying bacterial infections at the single cell level
Source: Chem Sci. 2022 Jun 29;13(27):8171–9. doi: 10.1039/d2sc02493d (PMC9278432; doi:10.1039/d2sc02493d)
Supplement: SC-013-D2SC02493D-s001 [file SC-013-D2SC02493D-s001.pdf]

Electronic Supplementary Material (ESI) for Chemical Science.
This journal is © The Royal Society of Chemistry 2022

Simultaneous Raman and Infrared spectroscopy: a novel combination for studying bacterial infections at the single cell level

Cassio Lima,‡[a] Shwan Ahmed,‡[a,d] Yun Xu,[a] Howbeer Muhamadali,[a] Christopher Parry,[b,c] Rachel J McGalliard,[b] Enitan D Carrol,[b] and Royston Goodacre*[a]

Results and discussion

Infrared spectroscopy

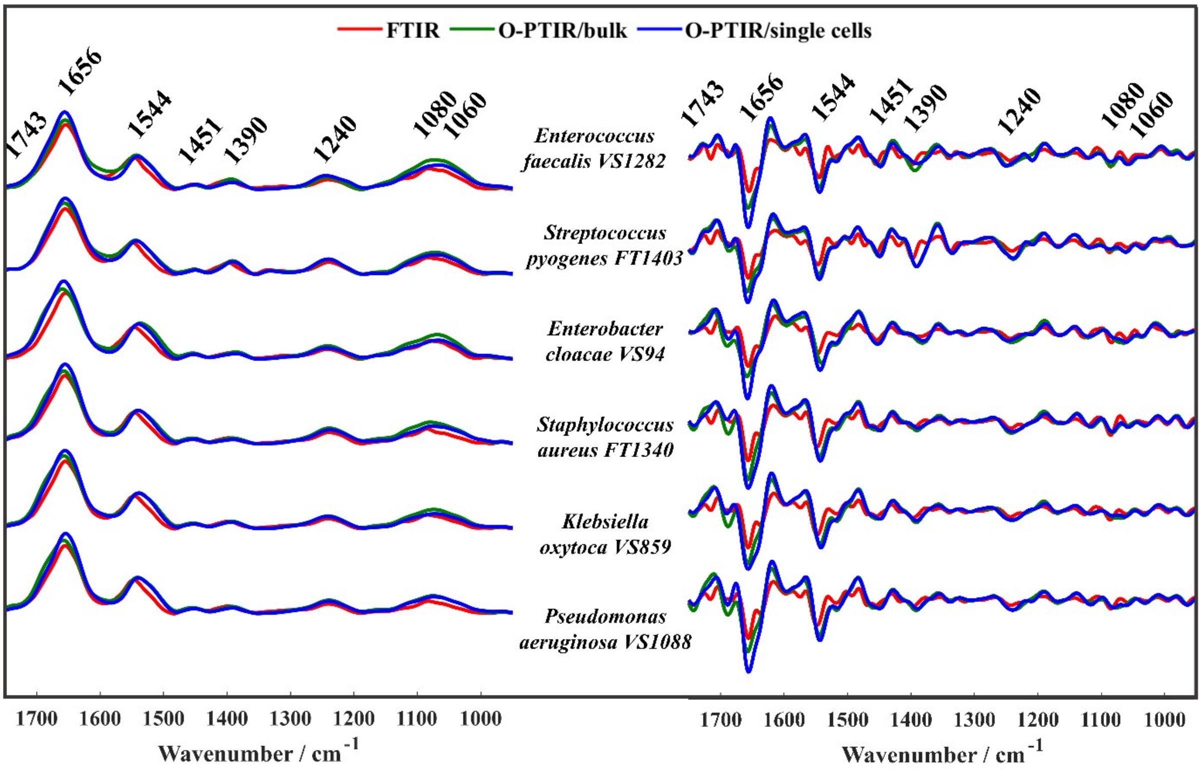

Figure S1. Average FTIR (red), O-PTIR/bulk (green), and O-PTIR/single cells (blue) spectra (left) and their second derivatives (right) collected from one strain of each bacterial species analysed in this study. The plots are offset for clarity. Key bands (in cm^{-1}) are highlighted.

Raman spectroscopy

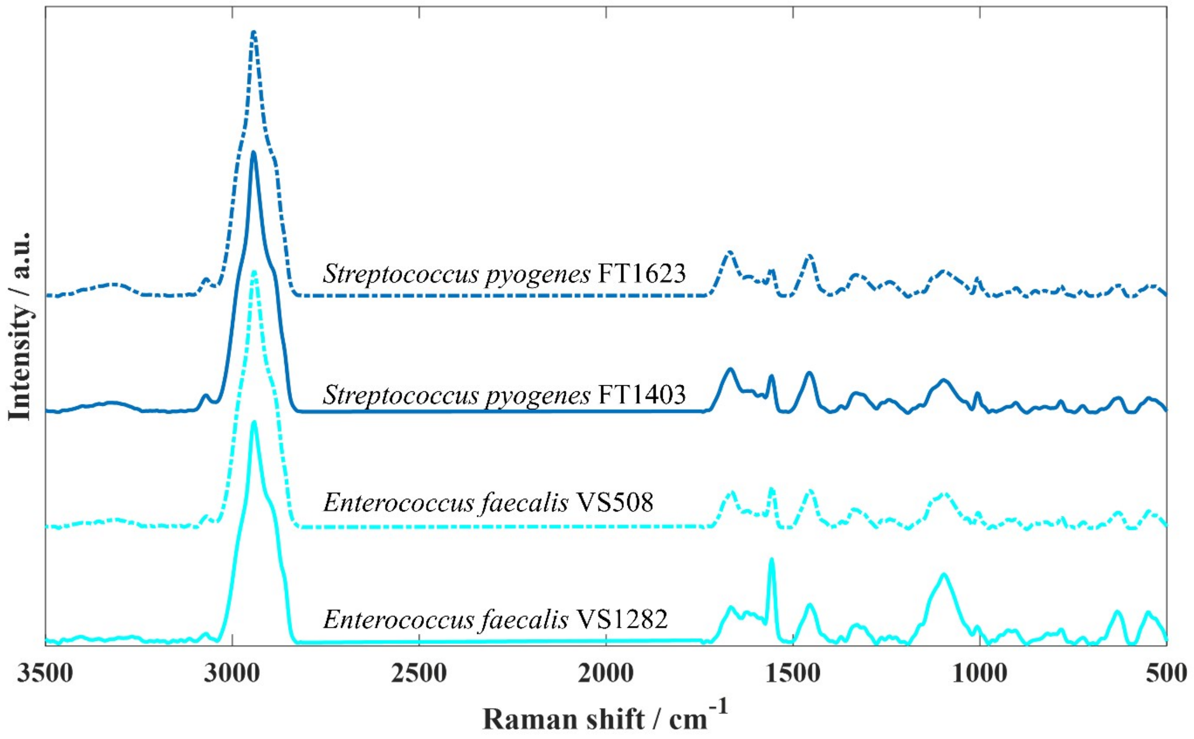

Figure S2. Average Raman spectra collected from single bacterial cells from the two strains of *S. pyogenes* and *E. faecalis*. Plots are offset for clarity.
